# The role of remote ischemic conditioning in ischemic stroke: neuroprotective mechanisms and future directions

**DOI:** 10.3389/fimmu.2026.1807395

**Published:** 2026-04-22

**Authors:** Tianrui Yu, Yanghang Chen, Yutao Lu, Fan Yu, Yuanyuan Xiang, Xinyue Kou, Hui Yang, Diyang Lyu, Moxin Wu, Xiaoping Yin, Zhiying Chen, Manqing Zhang

**Affiliations:** 1 Department of Neurology, Affiliated Hospital of Jiujiang University, Jiujiang, Jiangxi, China; 2 Jiujiang Clinical Precision Medicine Research Center, Jiujiang, Jiangxi, China; 3 Department of Neurology, The Second Affiliated Hospital of Nanchang University, Nanchang, Jiangxi, China; 4 College of Life Sciences, Nanchang University, Nanchang, Jiangxi, China; 5 Department of Neurology, Dongzhimen Hospital, Beijing University of Chinese Medicine, Beijing, China; 6 Jiangxi Provincial Key Laboratory of Cell Precision Therapy, School of Basic Medical Sciences, Jiujiang University, Jiujiang, Jiangxi, China

**Keywords:** remote ischemic conditioning, ischemic stroke, neuroinflammation, neuroprotection, combination therapy

## Abstract

Despite advances in vessel recanalization, ischemic stroke remains a leading cause of mortality, highlighting the need for comprehensive neuroprotective strategies such as remote ischemic conditioning (RIC). This review evaluates the multitargeted mechanisms of RIC, its progress in clinical translation, and the key factors determining its efficacy. In preclinical models, RIC exerts neuroprotection by modulating neuroinflammation, preserving the blood-brain barrier, and promoting angiogenesis and remyelination. Notably, it suppresses multiple programmed cell death pathways, including pyroptosis, apoptosis, ferroptosis, and disulfidptosis. However, analyses of recent high-quality clinical trials (e.g., SERIC-EVT, RESIST, and RICAMIS) reveal heterogeneous efficacy, indicating that clinical success is highly dependent on the specific execution protocol and successful cerebral reperfusion. Furthermore, critical patient-specific variables such as circadian rhythms, baseline systemic inflammation, and levels of both lipoprotein(a) and mean corpuscular hemoglobin (MCH) significantly influence therapeutic outcomes. Ultimately, while RIC is a highly translatable therapeutic strategy, its successful clinical application relies on the standardization of treatment protocols, the use of precision medicine to identify optimal responders, and its integration with existing therapies to maximize long-term stroke recovery.

## 1 Introduction

Ischemic stroke is caused by an interruption in cerebral blood flow induced by either thrombosis or embolism. It represents the second leading cause of death worldwide, accounting for 5.9 million deaths and 102 million disability-adjusted life years lost (1). To alleviate the disease burden of ischemic stroke, significant efforts have been made, including the development of intravenous thrombolytic therapy and mechanical thrombectomy; however, patient prognosis often remains poor (2). The primary reasons for this include delays in receiving endovascular therapy, post-treatment ischemia-reperfusion injury that damages endothelial and other cells, and persistent pathophysiological reactions following the stroke. Current data indicate that only 13.4% of stroke patients receive IVT or EVT, and only half of these individuals achieve functional independence at three months, highlighting the urgent need for new therapeutic strategies (3).

Remote ischemic conditioning (RIC), a safe and noninvasive emerging therapeutic approach, has been demonstrated in numerous studies to promote both neuroprotection and neurorepair following a stroke. The underlying mechanisms include the attenuation of neuroinflammation, inhibition of cell death, reduction of oxidative stress, protection of the blood-brain barrier (BBB), and promotion of remyelination. Moreover, because it is not restricted by a narrow therapeutic time window, RIC represents a novel therapeutic concept that could substantially improve the current outcomes for stroke patients (4).

This review first provides a comprehensive introduction to the concept of RIC and subsequently summarizes its therapeutic mechanisms. Furthermore, we review existing clinical trials, identify potential factors that may influence the efficacy of RIC, and discuss the feasibility of integrating RIC into combination therapies.

## 2 Overview of RIC

### 2.1 Definition and classification of RIC

RIC can be categorized into three subtypes based on the timing of its administration: (1) remote ischemic preconditioning, applied before an ischemic event to induce tolerance through brief ischemia and reperfusion at a remote site, thereby preventing ischemic damage; (2) remote ischemic perconditioning, applied during an ischemic event to activate protective mechanisms and minimize damage; and (3) remote ischemic postconditioning, applied after an ischemic event to induce protective factors that reduce reperfusion injury (5). For clarity, we will refer to these interventions collectively as RIC throughout this review. Notably, although the clinical presentations and treatment paradigms for acute and chronic ischemic cerebrovascular diseases differ significantly, both conditions severely impact brain health and contribute to cognitive impairment and other neurological deficits. Despite these differences, RIC has demonstrated significant therapeutic potential in both clinical scenarios. Consequently, in addition to the timing of initiation, the duration of therapy serves as an additional classification criterion for RIC. Beyond standard acute applications, long-term administration is formally recognized as chronic remote ischemic conditioning (CRIC) (6, 7). Current research on CRIC primarily focuses on its application in chronic cerebrovascular diseases (8). Although CRIC is currently less widely utilized than acute RIC protocols, its underlying mechanisms are closely linked to enhanced cerebral perfusion, highlighting its substantial potential in broader disease prevention.

### 2.2 Operation method of RIC

As previously discussed, RIC is a therapeutic technique involving short-term, repetitive occlusion of the brachial or femoral artery to induce multiple cycles of ischemia and reperfusion in the distal limb. However, intervention protocols differ significantly between clinical trials and animal studies. In clinical settings, a standard protocol involves alternating 5-minute cycles of ischemia and reperfusion for a total of five cycles, with cuff pressure typically set at 200 mmHg (or 20 mmHg above systolic blood pressure). This protocol is applied to either an upper or lower limb. However, no clinical studies have yet compared the efficacy of upper versus lower limb application, nor unilateral versus bilateral administration. Preclinical research in rhesus monkeys suggests that bilateral application appears to be more efficacious than unilateral treatment (9). Conversely, animal models typically utilize a three-cycle protocol comprising 10 minutes of ischemia followed by 10 minutes of reperfusion (10). Furthermore, the induction of RIC in certain experimental models can be highly invasive. Some researchers surgically expose the femoral artery by dissecting the anterior musculature. The artery is then alternately clamped and released using microvascular clips to achieve the conditioning effect (11). In contrast, a more widely adopted, non-invasive method employs a tourniquet around the hindlimb to occlude and subsequently release the femoral artery (12). Nevertheless, this procedure typically requires supplementary validation steps, such as using a pulse oximeter to confirm tissue hypoxia and palpating the skin of the lower limb to monitor temperature, to verify procedural success.

### 2.3 Potential risks of RIC treatment

**Figure 1:**
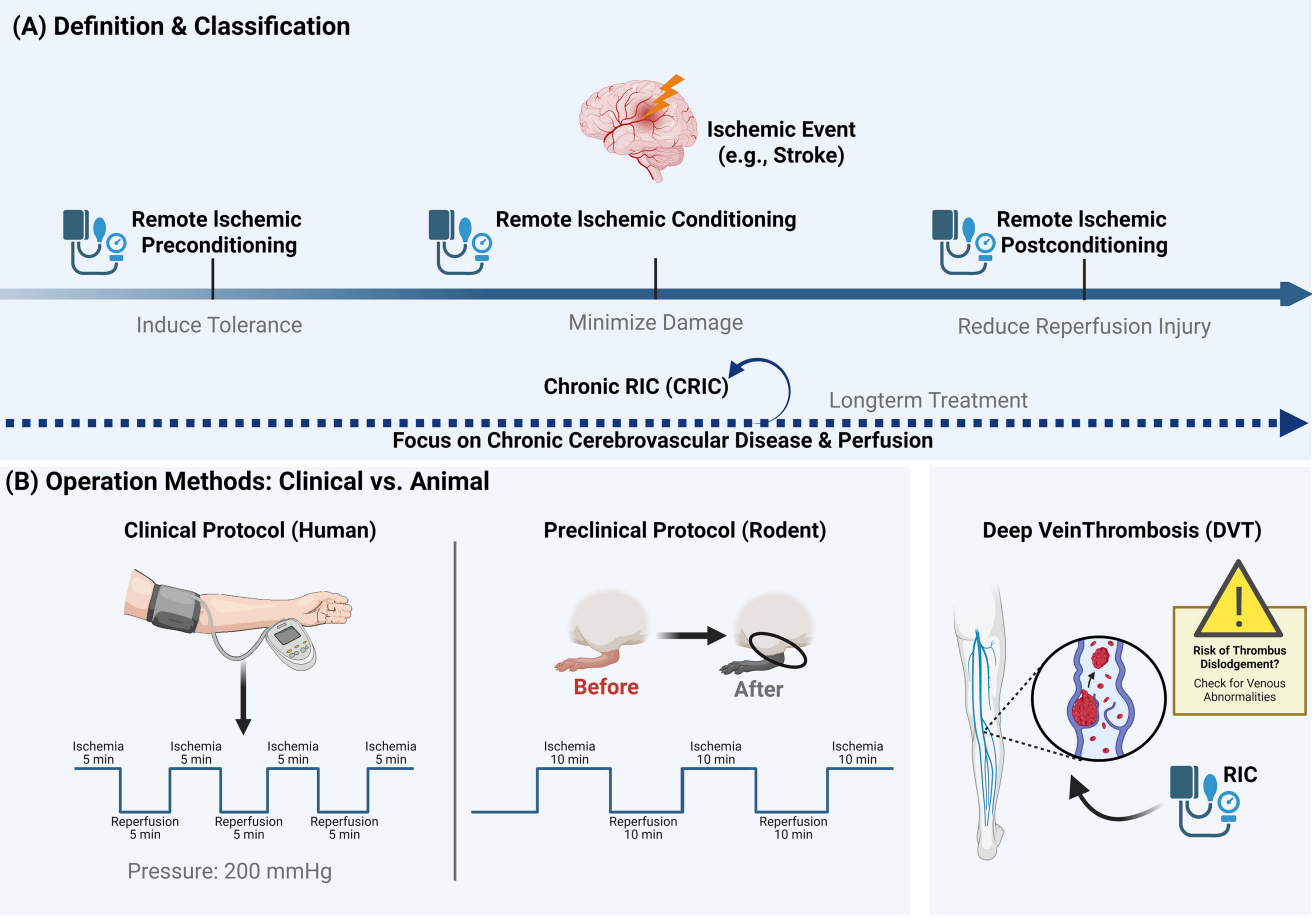
A comprehensive review of RIC **(A)** RIC can be divided into three types according to the action time. In addition, it can be divided into RIC and CRIC according to the duration of use. **(B)** Clinically, 200mmHg pressure is used for treatment, five minutes for inflation and five minutes for relaxation, and attention should be paid to deep vein thrombosis of lower limbs when using it. In animals, it usually takes 10 minutes to inflate and 10 minutes to relax.

Although RIC has demonstrated significant research value and therapeutic potential, limb-induced RIC treatment carries certain potential risks. Previous studies indicate that up to 50% of adults present with chronic lower limb venous abnormalities (13). Furthermore, patients with acute cerebrovascular diseases face an elevated risk of developing deep vein thrombosis (DVT) in the lower limbs (14). To date, no reports indicate that RIC treatment exacerbates lower limb venous thrombosis or induces thrombus dislodgement. Nevertheless, given the mechanical compression involved, it is crucial to consider the theoretical risks associated with the basic principles of RIC. Therefore, lower limb-induced RIC should be administered with caution in patients who have, or are at high risk for, lower limb venous thrombosis. In summary, a comprehensive vascular assessment of the patient’s lower limbs should be conducted prior to initiating RIC to confirm the suitability of this therapy (**Figure 1**).

## 3 Mechanism of RIC treatment

As previously delineated, the therapeutic efficacy of RIC is highly dependent on its execution protocol, which is broadly categorized into RIPreC, RIPerC, RIPostC, and CRIC. Although these distinct protocols share overlapping neuroprotective pathways, their specific molecular targets often vary depending on the timing of application relative to the ischemic event (15). For instance, RIPreC primarily induces endogenous ischemic tolerance prior to injury, whereas RIPostC focuses on mitigating reperfusion-induced cascades, such as reactive oxygen species (ROS) bursts and acute inflammation. To ensure clarity, the subsequent mechanistic discussions will explicitly identify the specific RIC protocols utilized in the cited preclinical and clinical studies.

### 3.1 Regulating neuroinflammation

The post-stroke inflammatory response, characterized by the activation of resident microglia and the infiltration of peripheral immune cells (such as neutrophils and T cells), critically exacerbates BBB dysfunction and neuronal death (16). Accumulating evidence indicates that RIC provides robust neuroprotection by spatiotemporally modulating these distinct immune populations.

After stroke, microglia are activated within a few hours and polarized into two different phenotypes: the pro-inflammatory M1 type and the anti-inflammatory M2 type (17–19). Emerging evidence suggests that RIC effectively modulates this key process. In murine middle cerebral artery occlusion/reperfusion (MCAO/R) models, RIPostC significantly reduces the expression of M1 markers (e.g., CD86 and iNOS) while upregulating M2 markers (e.g., CD206, Arg-1, and TGF-β) (20). Mechanistically, RIPostC achieves this by inhibiting NF-κB activation and promoting the nuclear translocation of PPARγ, thereby silencing pro-inflammatory cytokine production and alleviating neuroinflammation (21, 22).

As the earliest peripheral immune cells to infiltrate the ischemic brain, neutrophils correlate positively with infarct volume and functional impairment (23, 24). Research has demonstrated that RIPostC decreases the count of pro-inflammatory neutrophils among stroke patients (25). Furthermore, RIPostC effectively mitigates neutrophil-mediated damage by inhibiting NADPH oxidase (p47phox) activation and downregulating TNF receptor-associated factor 6 (TRAF6) expression. This signaling blockade suppresses ROS release from fMLP-activated neutrophils, ultimately reducing both the count and activity of pro-inflammatory neutrophils in the ischemic brain and peripheral circulation (26, 27).

In the later stages of stroke progression, T cells migrate from the peripheral circulation into the central nervous system. Studies indicate that the number of infiltrating T cells peaks between days 3 and 7 post-stroke, thereby exacerbating brain injury and neuroinflammation (28).

Notably, RIPreC and RIPostC exert distinct immunomodulatory effects on different T cell subsets, reflecting the time-dependent nature of their application. RIPostC, applied after ischemia onset, primarily activates neuroprotective regulatory T cells (Tregs) by decreasing the NAD^+^/NADH ratio, thereby enhancing the secretion of anti-inflammatory cytokines such as IL-10 (29–32). While Tregs provide protection against ischemic damage, the infiltration of cytotoxic CD8^+^ T cells significantly exacerbates brain tissue injury (33, 34). Conversely, RIPreC, applied prior to ischemia, modulates the peripheral immune system in a prophylactic manner. Studies have demonstrated that RIPreC reduces the circulating populations of CD8^+^ T cells and NKT cells (CD3^+^/CD161a^+^), increases non-inflammatory monocytes (CD43^+^/CD172a^+^), and modulates the production of cytokines such as IL-6 and TNF-α. Collectively, these effects attenuate the infiltration of CD8^+^ T cells into the brain parenchyma, thereby conferring neuroprotection (35).

**Figure 2:**
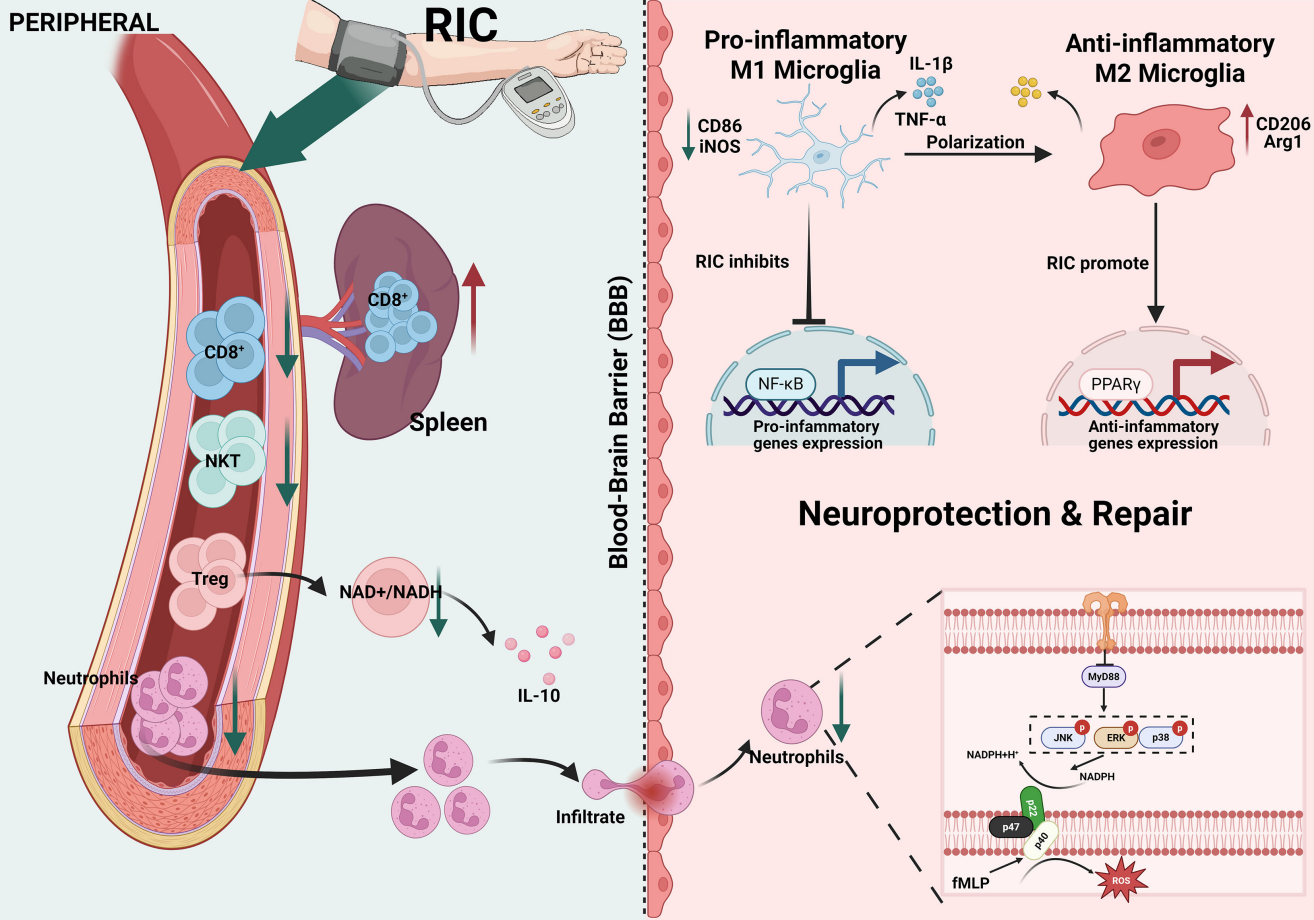
With the development of stroke, immune cells play a role successively, and RIC can improve neuroinflammation by regulating the activation and infiltration of these cells.

Previous studies have established that ischemic stroke induces splenic atrophy, which leads to a reduction in splenocyte counts, inhibition of T cell proliferation, and decreased secretion of inflammatory cytokines, ultimately resulting in systemic immunosuppression (36–38). To further elucidate the spleen’s role in RIC-mediated neuroprotection, Chen et al. demonstrated that RIPreC significantly increases the population of CD3^+^CD8^+^ cytotoxic T (Tc) cells within the spleen. Furthermore, their study revealed that splenectomy abrogates the neuroprotective efficacy of RIPreC in mice and reverses the beneficial shifts in peripheral blood immune cell composition (39). These findings underscore the divergent immunological effects of RIPreC and RIPostC likely reflect their distinct temporal windows of intervention. RIPreC preconditions the peripheral immune system prior to injury to attenuate subsequent T cell infiltration. In contrast, RIPostC, applied during the acute inflammatory phase, rapidly activates Tregs to counteract ongoing neuroinflammation. This temporal dichotomy suggests that these two strategies engage distinct yet complementary immunomodulatory pathways (**Figure 2**).

### 3.2 Regulating oxidative stress

Oxidative stress plays a pivotal role in the pathogenesis and progression of stroke, and the Nrf2 signaling pathway is identically crucial to the pathological process following ischemia. As a transcription factor that regulates the expression of antioxidant proteins, Nrf2 translocates from the cytoplasm to the nucleus under oxidative stress, where it binds to DNA promoters to initiate the transcription of antioxidant genes (40). Sun et al. established the MCAO mice models and treated the mice with RIPostC on days 1, 3 and 7. They observed that RIPostC markedly decreased oxidative stress by assessing the levels of glutathione/glutathione disulfide (GSH/GSSG) ratio, malondialdehyde (MDA), superoxide dismutase (SOD), and the total antioxidant capacity (TAC) in mice. In addition, they found that RIPostC significantly increased the mRNA expression and protein content of Nrf2 and HO-1 and reduced the expression of cleaved caspase-1, Keap1 and NLRP3, further confirming the neuroprotective effect of the Nrf2/HO-1 pathway after RIC (12). Together, these findings indicate that RIPostC exerts its antioxidant effects by both suppressing oxidative stress markers and upregulating the Nrf2/HO-1 pathway, thereby breaking the feedback loop between oxidative damage and neuroinflammation to confer neuroprotection.

### 3.3 Regulating cell death

#### 3.3.1 Pyroptosis

Pyroptosis is a form of programmed cell death that is intimately associated with neuroinflammation. Studies in MCAO/R rats demonstrate that the proportion of pyroptotic cells in the ischemic hemisphere peaks between 12 and 24 hours post-reperfusion, indicating a key role in ischemia-reperfusion injury (41). Mechanistically, the key features of pyroptosis include the formation of inflammasomes, the activation of proinflammatory cysteine proteases and gasdermin (GSDM) family proteins, and the subsequent release of proinflammatory cytokines. Following ischemic stroke, M1-type microglia predominate, and the activation of the TLR4/MyD88/NF-κB pathway promotes NLRP3 inflammasome assembly, leading to IL-1β release and microglial pyroptosis, which collectively exacerbate neuroinflammation (42, 43).

To investigate the effect of RIPostC on pyroptosis following stroke, Yu et al. conducted enzyme-linked immunosorbent assay (ELISA) analyses on serum samples from 42 patients with ischemic stroke at admission and three days post-treatment. The results demonstrated that levels of GSDMD, the terminal substrate in the pyroptosis pathway, positively correlated with the NIHSS score at admission, and that RIPostC treatment significantly reduced serum GSDMD levels in ischemic stroke patients (44). Furthermore, the cleavage of GSDMD generates an N-terminal fragment that binds to phosphoinositides in the plasma membrane, forming pores approximately 12–14 nm in diameter that directly mediate pyroptotic cell death. Remarkably, RIC treatment significantly reduced the density of GSDMD pores on microglial membranes within the ischemic penumbra (45). Finally, to elucidate the mechanism by which RIPostC inhibits pyroptosis, Yu et al. utilized high-throughput protein microarray screening and identified the HGF/ISG15 signaling axis as a key pathway through which RIC alleviates microglial pyroptosis following ischemic stroke. This finding was further validated in both an *in vitro* oxygen-glucose deprivation/reoxygenation (OGD/R) model and clinical serum samples (45). In summary, previous studies have elucidated the roles of HGF and ISG15 in regulating pyroptosis, and the anti-pyroptotic effect of RIPostC in ischemic stroke treatment appears to involve, at least in part, the HGF/ISG15 signaling pathway. This finding is consistent with earlier research and suggests that HGF may serve as a valuable biomarker for evaluating the therapeutic efficacy of RIPostC (46, 47).

#### 3.3.2 Apoptosis

Apoptosis is a form of programmed cell death involving endogenous pathways (e.g., mitochondria and endoplasmic reticulum) and exogenous pathways (e.g., death receptors) (48, 49). According to recent research, apoptosis plays a critical role in neuronal injury, the maintenance of neuronal function, and neurodevelopment (50, 51). In recent years, RIC has been shown to effectively attenuate apoptosis and exert significant neuroprotective effects, positioning it as a promising therapeutic approach. Research on endogenous pathways has demonstrated that RIPostC reduces apoptosis brought on by endoplasmic reticulum (ER) stress, thereby mitigating ischemic-reperfusion injury in mice. Subsequent studies further demonstrated that RIPostC exerts neuroprotective effects by inhibiting SERCA2-mediated ER stress and reducing apoptosis (52, 53). Additionally, studies have shown that RIPostC promotes the formation of mitochondrial-derived vesicles (MDVs) by reducing apoptosis-inducing factors such as AIF and EndoG from translocating from the mitochondria to the nucleus, thereby inhibiting apoptosis (54). Moreover, TLR4 is a signaling pathway that relative with apoptosis (55, 56). Recently, Lv et al. demonstrated that RIPostC and RIPerC significantly reduce mRNA expression and protein content of TLR4, TRAF6, MyD88, and NF-κB (57).

Regarding exogenous pathways, studies have shown that RIPerC exerts neuroprotective effects by upregulating the Bcl-2/Bax ratio through pre-activation of the Notch1 pathway and interaction with the NF-κB pathway, thereby reducing cerebral ischemia-reperfusion injury (58). Bcl-2 and Bax affect the activation of caspase by regulating the release of cytochrome C from mitochondria, ultimately leading to apoptosis (59–61). Despite these promising findings, current literature is heavily skewed toward RIPostC. Future research should comprehensively explore whether a synergistic application of RIPerC and RIPostC could provide a comprehensive neuroprotection.

#### 3.3.3 Ferroptosis

Ferroptosis is a distinct form of programmed cell death driven by iron-dependent lipid peroxidation. During ischemic stroke, cerebral ischemia induces intracellular iron accumulation, generating hydroxyl radicals via the Fenton reaction, which subsequently triggers lipid peroxidation and ultimately leads to ferroptosis (62, 63). Recently, Huang et al. demonstrated that RIPostC promotes the production of ketone bodies (KBs), increases cellular ATP levels, and reduces lactic acid accumulation. Additionally, RIPostC attenuates lipid peroxidation by regulating the expression of two critical regulators of ferroptosis: glutathione peroxidase 4 (GPX4) and acyl-CoA synthetase long-chain family member 4 (ACSL4). Moreover, RIPostC downregulates the expression of iron transport proteins, including the transferrin receptor (TFRC) and divalent metal transporter 1 (DMT1), thereby reducing intracellular total iron and ferrous ion levels (64). These findings suggest that RIPostC effectively mitigates ferroptosis, offering a novel therapeutic strategy for its inhibition. Future research should further delineate the precise signaling pathways through which RIPostC regulates ferroptosis to fully elucidate its neuroprotective mechanisms.

#### 3.3.4 Disulfidptosis

Disulfidptosis is a novel form of regulated cell death. Its occurrence is linked to the aberrant overexpression of SLC7A11, which leads to NADPH depletion, preventing the reduction of cystine to cysteine and resulting in the abnormal accumulation of intracellular disulfides. Consequently, the actin cytoskeleton undergoes contraction and disassembly due to aberrant disulfide cross-linking, ultimately triggering cell death (65, 66). While this form of cell death has been extensively studied in systems such as the respiratory, digestive, urinary, and reproductive systems, emerging evidence suggests that disulfidptosis may represent a promising therapeutic target for CNS disorders (67).

Liu et al. performed gene sequencing and experimental analyses in mice subjected to MCAO/R and identified a group of genes associated with the ability of RIPostC to mitigate disulfidptosis after stroke, including PRDX1, ACTB, CYFIP1, APBB1IP, and PGD. Among these, PRDX1 was identified as a key gene whose expression was significantly upregulated in the MCAO/R model and further enhanced following RIPostC treatment. Gene set enrichment analysis (GSEA) revealed that PRDX1 was closely associated with extracellular matrix–receptor interactions and calcium signaling pathways (68). However, previous studies have shown that PRDX1 plays a dual role in stroke. Specifically, following MCAO, PRDX1 promotes neuroinflammatory responses by activating the TLR4/NF-κB pathway, and its expression increases progressively over time (69). On the other hand, as an antioxidant protein, PRDX1 can initiate antioxidative responses by scavenging free radicals after stroke, thereby attenuating neuronal injury and preserving the integrity of the BBB (70). These findings suggest that the therapeutic effects of RIPostC may result from its ability to balance the opposing processes of antioxidation and neuroinflammation mediated by PRDX1.

#### 3.3.5 Efferocytosis

In the CNS, efferocytosis is primarily mediated by phagocytic cells such as microglia, macrophages, and neutrophils (71). As a process responsible for clearing apoptotic cells (ACs), efferocytosis is a fundamental biological mechanism for maintaining CNS homeostasis. Specifically, during CNS diseases such as cerebral ischemia, hemorrhage, or Alzheimer’s disease, ACs may fail to be efficiently removed. These ACs can undergo secondary necrosis, releasing damage-associated molecular patterns (DAMPs) that trigger a cascade of inflammatory responses. Through a well-coordinated, multi-step mechanism, phagocytes recognize the “find me,” “eat me,” and “don’t eat me” signals to promptly engulf ACs in the CNS, thereby preventing excessive inflammation and exerting neuroprotective effects (72, 73). Notably, a study performed whole-genome RNA sequencing on monocyte/macrophage populations isolated from both brain tissue and peripheral blood within five days after permanent focal cerebral ischemia in mice. The results revealed that, compared with blood-derived macrophages, multiple efferocytosis related genes (e.g., Gas6, C1q, ABCA1, CD36, and LAMP1) were upregulated in brain-resident macrophages. This finding not only underscores the complexity of the efferocytosis process but also identifies potential therapeutic targets among efferocytosis-associated genes in the CNS after stroke (74). Among these, CD36, a member of the class B scavenger receptor family, plays a key role in immune responses and efferocytosis. Previous studies have shown that CD36 remains highly expressed following stroke, promoting phagocytic uptake of myelin debris and alleviating neuroinflammation (75–77).

**Figure 3:**
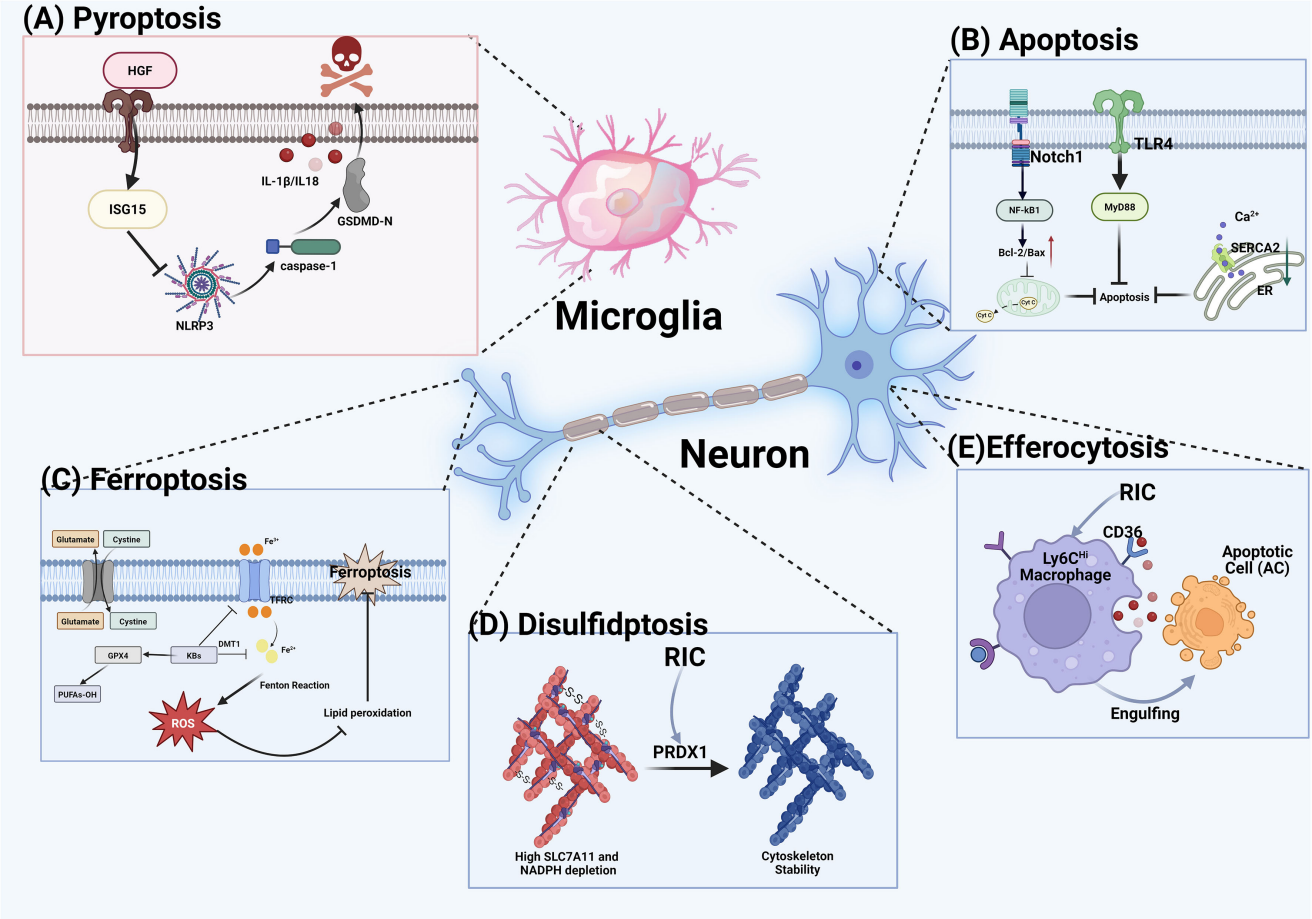
Mechanisms by which RIC modulates programmed cell death and clearance pathways. **(A)** Pyroptosis: RIC activates the HGF/ISG15 axis to inhibit GSDMD pore formation in microglia. **(B)** Apoptosis: RIC attenuates ER stress, balances Bcl-2/Bax, and promotes MDV formation to sequester apoptotic factors. **(C)** Ferroptosis: RIC reduces iron overload (TFRC/DMT1) and boosts antioxidant defense (GPX4) to prevent lipid peroxidation. **(D)** Disulfidptosis: RIC upregulates PRDX1 to counter disulfide stress and maintain cytoskeletal integrity. **(E)** Efferocytosis: RIC enhances CD36 expression on monocytes/macrophages, facilitating the clearance of cell debris and preventing secondary inflammation.

Interestingly, Ju et al. utilized a macrophage-specific CD36 deletion model to explore this pathway, demonstrating that the neuroprotective effect of RIPostC was abolished in the absence of CD36. However, because CD36 deletion itself fundamentally hampers baseline efferocytosis, the observed loss of protection cannot be attributed exclusively to the disruption of an RIPostC -mediated pathway. Instead, more supportive and direct evidence from their study lies in the dynamic changes in CD36 expression levels following RIPostC treatment. They found that RIPostC actively and selectively increased CD36 expression in Ly6C^Hi^ monocytes, but not in Ly6C^Low^ ones. A possible explanation is that Ly6C^Hi^ monocytes, known as inflammatory monocytes, rapidly migrate to inflamed sites and generally express low baseline levels of CD36 (thus relying heavily on RIPostC -induced upregulation), whereas Ly6C^Low^ monocytes inherently possess higher CD36 expression, making the effect of RIPostC less pronounced. Together, this robust expression data strongly confirms that RIPostC confers neuroprotection by actively upregulating CD36 to enhance efferocytosis (78) (**Figure 3**; **Table 1**).

**Table 1:** Summary of the forms of RIC-mediated cell death.

Type of cell death/clearance	Key mechanisms	Pathological role	Therapeutic modulation by RIC	Reference
Pyroptosis	Inflammasome (NLRP3) and GSDMD pores.	Severe neuroinflammation via releasing cytokines such as IL-1β.	Inhibits GSDMD pore formation via HGF/ISG15 axis.	(42, 45)
Apoptosis	Caspase activation and Bcl-2/Bax imbalance.	Core mechanism of neuronal loss.	Attenuates ER stress via SERCA2 inhibition; Inhibits TLR4/MyD88/NF-κB signaling; upregulates the Bcl-2/Bax ratio via Notch1 activation.	(52–54, 56, 57)
Ferroptosis	Iron overload and lipid peroxidation.	ROS-induced cellular damage	Reduces iron influx (TFRC/DMT1) and Boost ketone bodies generation.	(63, 64)
Disulfidptosis	high SLC7A11 levels, NADPH depletion and cytoskeleton collapse	Exacerbates structural neuronal injury.	Upregulates PRDX1 to stabilize cytoskeleton.	(67, 68)
Efferocytosis	Phagocytic clearance of ACs.	Inefficient clearance of ACs leads to secondary necrosis and the release of DAMPs, which triggers a cascade of inflammatory responses.	Enhances CD36 expression to accelerate ACs clearance.	(77, 78)

### 3.4 Alleviate excitotoxicity of glutamic acid in CNS

Following an ischemic stroke, hypoxia in the central nervous system disrupts ATP-dependent ion transport and neurotransmitter reuptake, leading to the excessive release and extracellular accumulation of glutamate. This overstimulates NMDARs and AMPARs, causing excessive Ca²^+^ influx, neuronal depolarization, and the activation of voltage-gated Ca²^+^ and Na^+^ channels. The resulting Ca²^+^ overload disturbs ion homeostasis and induces mitochondrial dysfunction, oxidative stress, and neuronal edema, ultimately culminating in cell death (79, 80).

Studies have shown that RIPreC significantly reduces glutamate levels in both the ischemic core and penumbra, mainly through the enhanced efflux of glutamate into peripheral blood. This effect depends on the glutamate transporter EAAT2 (81, 82). Further research indicates that RIPreC promotes the membrane translocation of EAAT1 and EAAT2 rather than increasing their expression, providing new evidence that RIC mitigates ischemic injury by facilitating glutamate clearance via EAATs (83).

### 3.5 Angiogenesis

The Notch signaling pathway is intimately associated with angiogenesis. The canonical Notch signaling pathway comprises its ligands (Jagged1, Jagged2, Delta-like1, Delta-like3, and Delta-like4), its receptors (Notch1, Notch2, Notch3, and Notch4), the CSL-DNA binding protein, and a series of downstream target genes (84). Recent studies demonstrate that RIPostC can enhance Notch1 pathway activity, promoting the development of microvessels in MCAO mice, thereby improving cerebral blood flow and facilitating neurological recovery (85). In addition, studies have shown that RIPostC treatment can up-regulate SDF-1α/CXCR4 signaling pathway from the third day to two weeks, thus improving brain injury and promoting angiogenesis (86).

### 3.6 Myelin regeneration

Oligodendrocytes in patients with cerebral ischemia are frequently damaged, resulting in demyelination (87–89). Previous studies indicate that oligodendrocytes play a crucial role in myelin regeneration and exhibit a close functional relationship with brain-derived neurotrophic factor (BDNF) (90). To investigate the relationship between RIPostC and remyelination, Li et al. administered RIPostC for 28 days in a rat model of chronic cerebral hypoperfusion, subsequently assessing cognitive function, oligodendrocyte counts, myelin density, and cell proliferation. Their results demonstrated that RIPostC inhibits oligodendrocyte apoptosis and promotes myelin formation in the corpus callosum while concurrently upregulating PTEN/Akt/mTOR signaling activity. Specifically, RIPostC exerts these remyelinating and anti-apoptotic effects by activating this signaling pathway following chronic cerebral hypoperfusion (91).

BDNF is an endogenous neurotrophin. Upon binding to its high-affinity receptor, TrkB (tropomyosin receptor kinase B), BDNF promotes neuronal survival, myelin regeneration, and synaptic plasticity (92–94). Given these critical roles, BDNF is regarded as a pivotal mediator of functional recovery in neurological diseases. Recent studies have demonstrated that RIPostC significantly enhances motor recovery in MCAO mice, exerting neuroprotective effects by regulating DNA 5mC methylation and upregulating BDNF mRNA and protein expression in the motor cortex (95).

### 3.7 Protect the blood-brain barrier

The BBB is a highly selective neurovascular unit composed of pericytes, brain endothelial cells (BECs), astrocytes, tight junctions, and a basement membrane (BM). It protects the CNS from circulating neurotoxins while regulating the transport of essential water-soluble nutrients and metabolites (96). However, in CNS disorders, the BBB dysfunction its structural integrity and normal function. This impairment allows previously restricted harmful substances to enter the CNS, breaking the immune-privileged status (97). This breach triggers a cascade of pathophysiological events, including immune cell infiltration, oxidative stress, and inadequate cerebral blood flow, thereby exacerbating brain damage. Consequently, protecting the BBB represents a promising therapeutic strategy.

Preclinical studies have shown that RIC mitigates BBB breakdown, alleviates cerebral edema, and reduces infarct volume. Immunofluorescence analyses reveal that RIPerC markedly restores the continuity of claudin-5 staining at the borders of cerebral endothelial cells. Interestingly, while RIPerC significantly increases occludin protein expression and decreases matrix metalloproteinase-9 (MMP-9) levels, the total protein expression levels of claudin-5 and ZO-1 remain unaltered (98). Furthermore, consistent with preclinical findings, clinical trials indicate that RIPostC reduces thrombolysis-exacerbated BBB damage following ischemic stroke by inhibiting the PDGF-CC/PDGFRα pathway (99). Notably, the protective effects of RIPostC extend to alleviating BBB dysfunction in models of Alzheimer’s disease. Ma et al. demonstrated that RIPostC enhances the expression of tight junction proteins, the pericyte marker PDGFRβ, and glucose transporter 1 (GLUT1) in APP/PS1 transgenic rats, further validating its barrier-preserving properties (100). The above literature shows that although the start time of treatment is different, both treatments at different times can alleviate the destruction of BBB, suggesting the potential of combined use of the two.

### 3.8 On the view of proteomics

Despite our growing understanding of RIC in recent years, many aspects of its underlying mechanisms remain unresolved. However, recent advances in proteomics have provided robust tools for elucidating the molecular mechanisms of RIPostC. For instance, a longitudinal study utilizing a macaque brain infarction model administered a five-week RIPostC intervention, employing weekly dynamic monitoring of plasma proteomic changes to explore the biological effects of RIC. Through GO, KOG, and KEGG pathway enrichment analyses, the study revealed that lipid metabolism regulation and anticoagulant responses exhibited significant alterations within the first two weeks of the initial RIPostC intervention. Additionally, complement activation and phagosome pathways became prominent after the third week of treatment, and by the end of the five-week intervention, the dynamic changes in protein regulation achieved a state of equilibrium (101). In addition, the same research team conducted further studies by performing proteomic analysis on blood samples from male patients before RIC, 5 minutes after RIC, and 2 hours after RIC. The results showed that RIC significantly inhibited the inflammatory response, particularly in the classical and lectin pathways, as well as the upregulation of complement precursor levels. At the same time, RIC significantly activated hemostasis balance (through stimulation of coagulation and fibrinolysis) and lipid metabolism regulation (through upregulation of ApoF). Therefore, based on these two proteomic analyses, it is evident that RIC exhibits superiority in its anti-inflammatory effects, especially with the significant increase in complement precursor (CPN) at 5 minutes after RIC, a phenomenon that was confirmed in both studies (102). These findings support the notion that healthy adults who undergo long-term RIC intervention may benefit from the induction of anti-inflammatory responses, potentially helping to prevent the occurrence of various vascular diseases. Furthermore, a study on the effects of RIPostC on the ischemic penumbra revealed that RIPostC facilitates the transition of the penumbra from a vulnerable to a tolerant state. RIPostC treatment significantly upregulated proteins associated with aminopeptidase activity, while downregulating proteins related to the cytoskeleton and glutamatergic synapses. These changes suggest that RIPostC can activate a series of neuroprotective pathways. In line with previous research, RIPostC also helps reduce glutamate accumulation. Notably, RIPostC induced a shift in the molecular weight of Map2 from 280 kDa to 70 kDa, a change that could potentially serve as a marker to distinguish between vulnerable and tolerant penumbra states (81, 103).

## 4 Progress in clinical research

### 4.1 Ischemic stroke

Numerous studies have investigated the therapeutic effect of RIPostC 6, 24 and 48 hours after the onset of an ischemic stroke (104–107). In addition, according to the etiology, clinical symptoms and treatment methods, stroke can be divided into anterior circulation stroke (ACS) and posterior circulation stroke (PCS). A trial for patients with ACS and PCS found that PCS patients are more likely to get a good functional outcome (mRS score) 90 days after RIPostC treatment, but this result may be affected by the imbalance of sample size between the two groups (108).

#### 4.1.1 Intravenous thrombolysis

Intravenous administration (IVT) of recombinant tissue plasminogen activator (rt-PA) within 3 to 4.5 hours following the onset of an ischemic stroke represents the most effective therapeutic approach for ischemic stroke (109). However, IVT is constrained by significant limitations. A considerable proportion of patients fail to achieve optimal recanalization, and the treatment can increase the risk of intracranial hemorrhage (ICH) while exacerbating BBB disruption (110). Interestingly, Hoda et al. demonstrated that combining RIPerC with rt-PA restored cerebral blood flow (CBF), reduced infarct volume, and increased Akt phosphorylation within the ischemic hemisphere of MCAO mice (111). It is worth noting that after eMCAO, the level of Akt rises transiently, thereby suppressing nitrative stress. RIPerC prolongs Akt activation while inhibiting PI3K (a major upstream kinase and Akt activity regulator), indicating that the PI3K/Akt signaling pathway is a critical pathway through which RIPerC exerts its neuroprotective effects. Furthermore, a key synergistic mechanism between RIPostC and IVT lies in preserving the BBB. While rt-PA can worsen BBB disruption by activating the PDGF-CC/PDGFRα pathway, RIPostC directly counteracts this. He et al. demonstrated that RIPostC lowers circulating PDGF-CC levels, thereby preventing PDGFRα activation in ischemic brain tissue. By mitigating rt-PA-induced BBB leakage, hemorrhagic transformation, and inflammation, RIPostC significantly enhances the safety of delayed thrombolysis (99).

Studies have shown that combining RIPerC with rt-PA can improve cerebral blood flow, reduce infarct volume (111, 112). In the meanwhile, He et al. assessed the effectiveness and safety of RIPostC in combination with IVT. The findings showed no discernible variations (113). Nevertheless, in the SERIC-IVT study initiated by Yang et al., 558 patients were included in the study (RIPostC parameter was set to unilateral upper limb; The pressure is 200mm Hg, twice a day for 7 days). As the largest randomized controlled clinical trial of IVT patients, the results show that it is safe for ischemic stroke patients receiving IVT to receive RIPostC twice a day for 7 days. However, RIPostC has not significantly improved the incidence of excellent functional outcomes in ischemic stroke patients receiving IVT (114).

#### 4.1.2 Endovascular thrombectomy

Endovascular thrombectomy (EVT) is one of the treatment methods for acute ischemic stroke due to large vessel occlusion. However, in reality, patients who undergo successful recanalization with EVT often experience poor outcomes (115). Regarding the efficacy of RIC in EVT-treated patients, recent high-quality clinical trials have yielded seemingly contradictory yet complementary evidence (116, 117). Specifically, the SERIC-EVT trial demonstrated that among patients who underwent thrombectomy for anterior circulation large vessel occlusion, a 7-day course of RIPreC and RIPostC significantly increased the proportion of patients achieving functional independence at 90 days while maintaining a favorable safety profile. This finding supports RIC as a broadly applicable neuroprotective strategy following EVT. In contrast, a *post-hoc* analysis of the RESIST trial failed to demonstrate an overall benefit of RIPreC and RIPostC in the EVT-treated population, indicating instead that therapeutic efficacy might be highly dependent on successful recanalization.

#### 4.1.3 Physical exercise

Physical exercise is a promising rehabilitation strategy that has demonstrated significant benefits in stroke recovery (118–120). Extensive evidence indicates that physical exercise promotes functional recovery and reduces stroke-related complications by stimulating angiogenesis, enhancing neuronal repair, and restoring the integrity of the BBB (121–123). However, physical exercise therapy presents certain clinical limitations. Notably, evidence indicates that initiating exercise within 24 hours of stroke onset may negatively impact patient outcomes (124). In contrast, the therapeutic efficacy of RIPostC is not strictly constrained by a narrow time window, allowing it to serve as a versatile, time-independent adjunct to exercise therapy (125). Recently, Geng et al. compared the mRNA and protein levels of neuroplasticity factors, synaptic proteins, angiogenic factors, and regulatory molecules (e.g., BDNF, TrkB, CREB, and HIF-1α) in mice following either exercise or RIPostC therapy. Compared to physical exercise, RIPostC proved more effective at reducing acute ischemic infarct volume and the severity of neurological deficits, while concurrently enhancing neuroplasticity, angiogenesis, and synaptogenesis (126). Further exploring the synergistic potential of these interventions, recent studies have shown that combining RIPostC with physical exercise significantly increases the mRNA and protein expression of SYN, PSD-95, and BDNF in MCAO mice, while simultaneously inhibiting NLRP3 inflammasome activation (127). Overall, current research focuses on combining physical therapy with ischemic therapeutic strategies to optimize treatment timing or explore the synergistic application of both to maximize rehabilitation outcomes.

#### 4.1.4 Other treatment

Medical engineering technology and traditional Chinese medicine can work together. This combination enhances the therapeutic efficacy of RIC. Medical engineering optimizes the implementation and evaluation of RIPostC. For example, tissue reflection sensors enable individualized pressure regulation. Near infrared II fluorescent probes and online electrochemical systems monitor brain blood flow. They also track dynamic changes in ascorbic acid. These tools help researchers understand the neuroprotective mechanisms of RIPostC (128–131). On the other hand, the combination therapy of traditional Chinese medicine and RIPostC shows great potential. When Paeoniflorin combined with RIPostC to treat I/R injury, studies demonstrated that the combination therapy, compared with RIPostC or paeoniflorin alone, markedly inhibited neutrophil accumulation. Additionally, studies have shown that combination therapy inhibits NADPH oxidase activation in neutrophils and the MyD88-TRAF6-p38 MAPK signaling pathway, leading to enhanced therapeutic effects (132).

While the present review focuses primarily on RIC, it is worth noting that the concept of “conditioning” extends beyond localized ischemia. Intermittent Hypoxia Conditioning (IHC), achieved through intermittent inhalation of hypoxic air, represents another non−invasive strategy aimed at harnessing endogenous tolerance mechanisms against ischemic injury. Although the two approaches differ in their initial triggers, they converge on common downstream effectors, including stabilization of hypoxia-inducible factors (HIFs), modulation of inflammatory cascades, and protection of the BBB (133). Crucially, the successful execution of these therapies hinges on the fundamental distinction between continuous and intermittent protocols. Continuous or sustained hypoxia/ischemia imposes a severe pathological stress that leads to detrimental outcomes, including severe energetic collapse and irreversible white matter damage (134). In contrast, intermittent conditioning leverages the biological principle of hormesis. Mechanistically, the brief reperfusion or reoxygenation intervals unique to intermittent protocols are indispensable. They generate controlled bursts of ROS that act as vital secondary messengers to upregulate antioxidant capacity, thereby averting the pathological damage associated with continuous exposure (135). Ultimately, a comprehensive understanding of these broader conditioning paradigms provides critical mechanistic insights that can inform the future optimization of RIC protocols and facilitate the development of synergistic combination strategies.

## 5 Factors that may affect the curative effect of RIC

### 5.1 Circadian rhythm

Although preclinical and clinical data for RIPostC in stroke treatment are promising, its therapeutic efficacy in clinical practice remains inconsistent. One possible reason is that the circadian rhythm is neglected in the experimental design. A critical challenge exists, most successful animal studies of RIPostC are conducted during the daytime, which corresponds to the rest (inactive) phase for nocturnal rodents. In contrast, the incidence of human stroke peaks during the daytime, our active phase. This discrepancy means that a therapy proven effective in animals might be applied during the opposite circadian phase in humans.

A recent study by Licastro et al. directly tested this hypothesis. They found that RIPostC was only effective in reducing infarct volume and improving functional recovery when administered during the inactive phase of mice; it had no effect during their active phase. This defines a precise therapeutic time window for RIPostC. Mechanistically, RIPostC specifically reduced levels of the core clock protein PER2 and increased levels of the neuroprotective protein nNOS, but only in the inactive phase. Critically, pharmacological inhibition of nNOS abolished both the neuroprotective effects of RIPostC and its regulation of PER2. This indicates that nNOS activation is a prerequisite for PER2 downregulation and the subsequent neuroprotection, a mechanism exclusively triggered during the inactive phase (136).

In summary, circadian rhythm critically affects the efficacy of RIPostC and may explain inconsistent clinical outcomes. Future studies should consider the time of stroke onset when evaluating RIC therapy.

### 5.2 Lp(a)

Elevated lipoprotein(a) [Lp(a)] is an established risk factor for atherosclerosis and is associated with prognosis in patients with symptomatic ICAS. Emerging evidence suggests that Lp(a) levels may also influence the efficacy of CRIC (137). A recent *post-hoc* analysis of the RICA trial demonstrated that in patients with symptomatic ICAS, baseline Lp(a) levels >17.4 mg/dL were associated with an increased risk of stroke recurrence (adjusted HR 1.38). Notably, the efficacy of CRIC varied by Lp(a) levels: in patients with elevated Lp(a) (>17.4 mg/dL), the stroke incidence was 16.7% in the CRIC group versus 22.6% in the control group (adjusted HR 0.67), whereas no significant protective effect was observed in those with lower Lp(a) levels (≤17.4 mg/dL) (138).

These results suggest that Lp(a) level may be used as a biomarker to predict the efficacy of CRIC. However, the role of Lp(a) in the treatment of stroke by CRIC needs to be studied.

### 5.3 MCH

MCH plays a key role in many physiological and pathological states. Recent studies show that MCH may be one of the important factors affecting the curative effect of RIPostC, especially in patients of different age groups. Studies have shown that MCH is closely related to the neuroprotective effect of RIPostC treatment, and in patients over 60 years old, the high MCH group shows more obvious therapeutic effect. This result suggests that MCH may enhance the neuroprotective effect of RIPostC by affecting the oxygen carrying capacity of red blood cells, antioxidant response and microcirculation regulation. In addition, it is worth noting that other red blood cell indexes (such as red blood cell count, HCT, MCV, HB, MCHC) have not shown similar effects (139).

### 5.4 Inflammation

**Figure 4:**
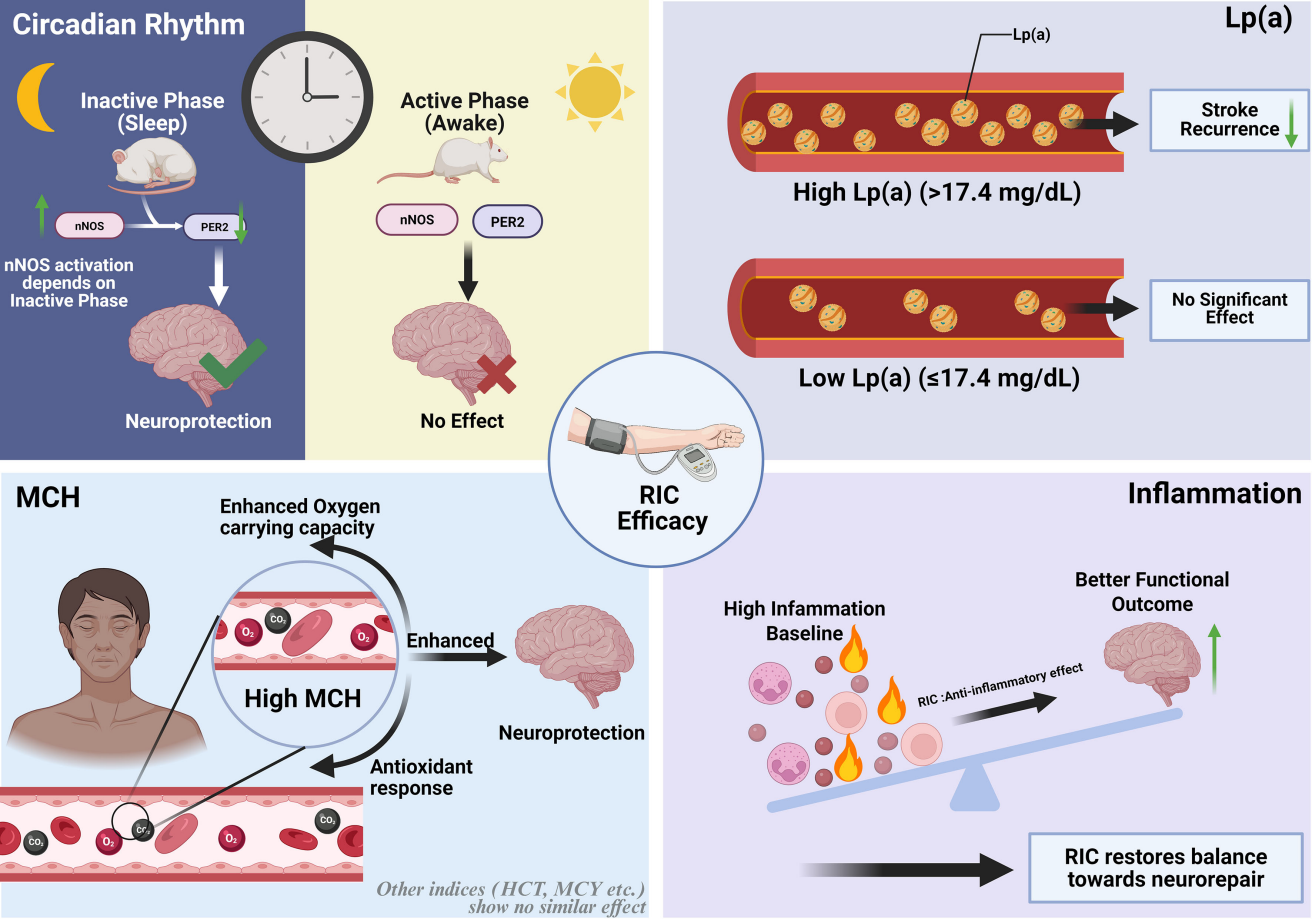
Factors that may affect the curative effect of RIC include: circadian rhythm, Lp(a), MCH, inflammation.

More and more evidence shows that inflammatory reaction plays a double-edged sword role in ischemic stroke, involving nerve repair and nerve injury (140). Previous studies have shown that anti-inflammatory effect is one of the mechanisms of neuroprotective effect of RIPostC. However, the effects of inflammatory indicators (such as neutrophil-lymphocyte ratio (NLR), platelet-lymphocyte ratio (PLR) and systemic immune inflammatory index (SII)) on the efficacy of RIPostC intervention have not been studied. The research results of Wang et al. show that RIPostC can significantly improve the 90-day functional outcome of patients with high or low inflammation. An interesting trend is that patients with high baseline inflammation levels (such as high NLR and high SII groups) get more absolute benefits from RIPostC treatment than patients with low inflammation levels (141). It is suggested that patients with high inflammatory reaction may be the potential better beneficiaries of RIPostC, and the improvement of neurological function may be partly due to the regulatory effect of RIPostC on systemic inflammation (**Figure 4**).

## 6 Hypotheses regarding RIC efficacy

Currently, numerous large-sample, high-quality clinical trials (including RICA, SERIC-EVT, and RESIST) have been conducted to investigate the therapeutic efficacy of RIC. However, these trials have yielded highly heterogeneous findings that warrant further discussion, highlighting the urgent need for a critical systematic analysis of this evidence base. Therefore, to systematically compare the discrepancies between existing high-quality clinical studies and identify the key factors driving divergent outcomes, we have compiled **Table 2** for readers’ reference, and additionally present our perspectives on future research directions. Through systematic analysis of these clinical trials and the available evidence, we propose that RIC treatment regimen parameters may be the key determinant of divergent clinical outcomes (114, 142). Notably, findings from the SERIC-EVT trial indicate that the therapeutic efficacy of combined RIPreC and RIPostC is highly dependent on successful vessel recanalization (117). This observation is further corroborated by a *post-hoc* analysis of the RESIST trial, which demonstrated that the neuroprotective benefits of RIPreC and RIPostC are predominantly manifested only in patients who achieve complete reperfusion after IVT or EVT (116). Mechanistically, this finding aligns with the core mechanistic principle of RIPostC: as RIPostC primarily targets reperfusion-induced injury cascades (e.g., ROS bursts and secondary BBB disruption), its therapeutic potential is inherently dependent on the restoration of cerebral blood flow. Furthermore, accumulated preclinical and clinical evidence consistently supports the promising therapeutic potential of CRIC. Taken together, findings from these high-quality clinical trials collectively suggest that long-term treatment regimens, high-intensity RIC administration, and intact cerebral perfusion in patients are the core factors mediating the clinical benefits of RIC, which warrant further validation in future clinical studies.

**Table 2:** Existing RIC high-quality research and related analysis.

Clinical trial	Population	Study design	Protocol	Key primary	Factors potentially	References
SERIC-IVT	Ischemic stroke, with IVT	Multicenter, RCT, Sham -controlled	Post-IVT (mean 13.5h after onset), 7 days, Unilateral, 200 mmHg	RIPostC combined with IVT is safe. But it did not significantly improve the functional outcome.	Milder stroke severity, Delayed initiation of RIC (mean 13.5h), Unilateral limb stimulus	(114)
SERIC-EVT	Ischemic stroke, with EVT	Multicenter, RCT, Sham-controlled	Post-EVT (within 24h), 7 days, Unilateral, 200 mmHg	RIPostC combined with EVT improved the functional outcome.	High rate of successful recanalization	(117)
RESIST	Ischemic stroke or ICH		7 days, Unilateral, 200 mmHg	RIPreC and RIPostC did not significantly improve the functional outcome.	Low treatment compliance, Low median NIHSS score, High reperfusion treatment rate (contradictory to other trials)	(143)
RICAMIS	Ischemic stroke	multicenter, open-label, blinded-end point, randomized clinical trial	10–14 days, bilateral, 200 mmHg	RIPostC improves the possibility of excellent neurological function at 90 days.	Smoke attenuate RIC efficacy, the treatment intensity is higher, the target population had a stroke within 48 hours.	(104, 144)
RICA	ICAS	a multicentre, randomised, double-blind sham-controlled trial in China	1 year, bilateral, 200 mmHg	In the subgroup of patients with good compliance, CRIC showed significant benefits.	Low compliance, The stroke risk of ICAS itself naturally decreases with time.	(145)
REPOST	Ischemic stroke	a randomized single-blind placebo-controlled clinical trial,	4 days, 200 mmHg	There was no significant improvement in infarct size or clinical outcome after RIPostC treatment.	Small infarct size, short intervention time	(146)

## 7 Conclusions and perspectives

Remote ischemic conditioning is a safe and highly translatable therapeutic strategy for ischemic stroke. It exerts multifaceted neuroprotective and neurorestorative effects. These effects occur through the modulation of neuroinflammation and diverse programmed cell death pathways. The treatment also preserves BBB integrity, promotes angiogenesis, and enhances remyelination. The specific timing of intervention is critical. However, this review has certain limitations. First, this article extensively describes the neuroprotective effects of RIPostC. It fails to compare RIPostC with other treatments. It also lacks a summary of mechanistic differences. This omission largely occurs because current research focuses heavily on RIPostC. Second, RIC is an effective treatment for multiple diseases. However, this review focuses solely on ischemic stroke.

In summary, current research indicates a clear trend. The therapeutic efficacy of RIC strictly depends on successful cerebral reperfusion. The combined application of RIPreC and RIPostC shows significant benefits. These benefits appear specifically in patients with successful vessel recanalization. Furthermore, researchers must consider the physiological state of patients. Key variables include circadian rhythms and baseline inflammation levels. Lipoprotein(a) and mean corpuscular hemoglobin are also critical. These parameters significantly influence therapeutic outcomes.

Looking forward, the successful clinical implementation of RIC depends on these core elements. First, researchers must establish standardized treatment protocols. These protocols must target specific clinical scenarios. Second, the medical community needs a reliable biomarker. This biomarker will confirm the therapeutic efficacy of RIC. Third, future studies should optimize combination therapy regimens. Finally, further mechanistic research on CRIC is necessary. This research will clarify its role in long term neurorestorative effects.
